# Risk Factors and Clinical Predictors of Drug-Induced Hepatotoxicity in Patients Receiving First-Line Anti-tuberculosis Therapy

**DOI:** 10.7759/cureus.110655

**Published:** 2026-06-11

**Authors:** Hammad Khan, Momna Arif, Farzana Aftab, Amjid Khan, Ibrahim Allah Diwaya, Syed Haider Farooq Sherazi, Muhammad Younas Ali, Muhammad Wasil, Ziadullah Khan, Naqeeb Ullah, Najeeb Ullah Noraiz

**Affiliations:** 1 Intensive Care Unit, Hayatabad Medical Complex, Peshawar, PAK; 2 Acute Internal Medicine, Midland Metropolitan University Hospital, Smethwick, GBR; 3 Medicine, Lady Reading Hospital, Peshawar, PAK; 4 Obstetrics and Gynaecology, Naqaish Medicare, Islamabad, PAK; 5 Emergency Medicine, Tipperary University Hospital, Clonmel, IRL; 6 General Practice, Dar Al Tadawi Clinics, Jeddah, SAU; 7 Internal Medicine, Hayatabad Medical Complex, Peshawar, PAK; 8 Pediatric Intensive Care Unit, Al-Adan Hospital, Hadiya, KWT; 9 Medicine, Kuwait Teaching Hospital, Peshawar, PAK; 10 Internal Medicine, Lady Reading Hospital, Peshawar, PAK; 11 Medicine, Khyber Medical University, Peshawar, PAK

**Keywords:** anti-tuberculosis therapy, drug-induced hepatotoxicity, prealbumin, risk factors, serum albumin, tuberculosis

## Abstract

Background: Drug-induced hepatotoxicity is a major adverse effect of first-line anti-tuberculosis (TB) therapy that can compromise treatment safety and outcomes.

Objective: The objective of this study was to identify and evaluate clinical, laboratory, and nutritional factors associated with drug-induced hepatotoxicity in patients receiving first-line anti-TB therapy.

Materials and methods: A hospital-based prospective observational study was conducted at Lady Reading Hospital, Peshawar, Pakistan, from January 2023 to June 2024. A total of 220 adult patients with confirmed pulmonary or extrapulmonary TB receiving first-line anti-TB therapy comprising isoniazid, rifampicin, pyrazinamide, and ethambutol were included. Data included demographics, comorbidities, liver function tests (alanine aminotransferase (ALT), aspartate aminotransferase (AST)), and nutritional biomarkers (serum albumin, prealbumin, and total protein). Follow-up liver function tests and nutritional assessments were performed weekly during the first month and subsequently at week 6 and week 8. Drug-induced hepatotoxicity was defined as ALT/AST levels greater than three times the upper limit of normal (ULN) with symptoms or greater than five times ULN without symptoms. Statistical analysis included chi-square test, independent t-test, and multivariate logistic regression.

Results: Drug-induced hepatotoxicity occurred in 38 of 220 patients (17.27%). Based on the severity of liver enzyme elevation and clinical presentation, 20 (9.09%) had mild hepatotoxicity, 12 (5.45%) had moderate hepatotoxicity, and six (2.73%) had severe hepatotoxicity. Comparisons were performed using pretreatment clinical, laboratory, and nutritional parameters measured before initiation of therapy, and outcomes were assessed during follow-up. Significant associated factors included age >45 years (47.37% vs 25.82%; adjusted OR (aOR) 2.15), female sex (57.89% vs 42.86%; aOR 1.87), alcohol use (31.58% vs 9.89%; aOR 3.42), and viral hepatitis (21.05% vs 3.85%; aOR 5.28). Nutritional factors associated with hepatotoxicity included low serum albumin (<3.5 g/dL; aOR 2.12) and low prealbumin (<20 mg/dL; aOR 2.45). Liver function test parameters measured at the time of hepatotoxicity diagnosis during follow-up were significantly higher in affected patients compared with those without hepatotoxicity.

Conclusion: Drug-induced hepatotoxicity during first-line anti-TB therapy is associated with specific clinical, laboratory, and nutritional risk factors that can help identify patients at higher risk for developing this adverse outcome. Future research should focus on validating these predictors in larger populations and developing targeted monitoring and prevention strategies to improve treatment safety and outcomes.

## Introduction

One of the most clinically important adverse effects of anti-tuberculosis (TB) therapy is drug-induced hepatotoxicity, which occurs in particular during first-line multidrug regimens [1]. TB has been a significant health issue in the world, especially in developing nations [2]. The most common first-line regimen, which is made of isoniazid, rifampicin, pyrazinamide, and ethambutol, is highly effective but is associated with well-developed hepatotoxicity, which may weaken patient safety, cause interruption of the treatment, and increase the chances of developing drug resistance [3,4].

Mechanisms of hepatotoxicity are multifactorial and entail complicated interactions of drug metabolism, host genetic susceptibility, and environmental factors [5]. The most commonly implicated ones are isoniazid and pyrazinamide, as they mediate their hepatocellular injury via production of toxic metabolites, and rifampicin may cause liver injury by boosting production of hepatic enzymes and creating more reactive intermediates [6,7]. Clinical manifestations range from asymptomatic elevation of liver enzymes to severe hepatitis, acute liver failure, and, in rare cases, mortality [8].

There are a number of patient- and treatment-related risk factors that elevate the risk of hepatotoxicity. They encompass old age, female sex, malnutrition, alcoholic drinks, pre-existing liver disease, viral co-infection of hepatitis, and genetic polymorphism involving drug metabolism [9,10]. Moreover, other comorbidities like diabetes mellitus and HIV infection might also predispose patients to liver injury. Timely detection of high-risk patients is thus important so that the continuation of therapy may be safe [11,12].

Although these risk factors have been identified, hepatotoxicity incidence varies widely among diverse populations, and region-specific data are not available, especially in resource-limited settings. Monitoring strategies and reduction in treatment-related complications should be optimized by evaluation of clinical predictors and risk factors of the local population. Our goal was to identify and evaluate the risk factors and clinical predictors associated with drug-induced hepatotoxicity in patients receiving first-line anti-TB therapy.

## Materials and methods

Study design and setting

This was a prospective observational study conducted at Lady Reading Hospital, Peshawar, Pakistan, a significant tertiary care facility with specialized diagnostic and therapeutic capabilities, over 18 months from January 2023 to June 2024. The study was approved by the Institutional Review Board of Lady Reading Hospital (reference number: 1029/LRH/MTI; dated December 12, 2022) and complied with the Declaration of Helsinki and preserved confidentiality. Every participant provided written informed consent.

Study population

To evaluate drug-induced hepatotoxicity and related clinical predictors, all eligible patients with pulmonary or extrapulmonary TB who were started on first-line anti-TB medication were sequentially recruited and monitored. The inclusion and exclusion criteria for participant selection are summarized in Table 1.

**Table 1:** Inclusion and exclusion criteria Patients with stable chronic viral hepatitis (HBV/HCV) and non-dependent alcohol use without advanced liver disease were included, as these variables were evaluated as potential predictors of drug-induced hepatotoxicity. Patients with alcohol dependence, alcohol use disorder requiring medical treatment, or evidence of alcoholic liver disease were excluded. HBV: hepatitis B virus; HCV: hepatitis C virus

Category	Criteria
Inclusion Criteria	Age ≥18 years
	Confirmed diagnosis of tuberculosis (pulmonary or extrapulmonary)
	Initiation of first-line anti-tuberculosis therapy
	Provided written informed consent
	Willingness to comply with follow-up visits
Exclusion Criteria	Pre-existing cirrhosis
	Decompensated chronic liver disease
	Baseline liver enzymes >2× upper limit of normal
	Use of second-line anti-tuberculosis drugs
	Concurrent use of other hepatotoxic medications
	Pregnant or lactating women
	Advanced alcohol-related liver disease or alcohol dependence
	Severe active hepatitis with hepatic dysfunction

Sample Size

The sample size was calculated using the formula for prevalence studies [13]: $n=\frac{Z^2 \times p \times (1-p)}{d^2}$. Where n represents the required sample size, Z is the standard normal variate at a 95% confidence level (1.96), p is the anticipated prevalence of drug-induced hepatotoxicity (assumed to be 15%), and d is the margin of error (5%). The calculated sample size was approximately 196 participants. To account for possible dropouts, incomplete data, and loss to follow-up, the sample size was increased by approximately 10-15%, resulting in a final sample size of 220 patients.

Data collection

A standardized proforma developed and approved by the study team in consultation with clinical and research experts was used for data collection. Baseline demographic characteristics, clinical history, comorbidities (including diabetes mellitus and chronic viral hepatitis), social variables (including smoking status and alcohol use), and nutritional biomarkers (serum albumin, prealbumin, and total protein levels) were recorded. Alcohol use was defined as self-reported consumption of one or more alcoholic drinks per month during the six months preceding initiation of anti-TB therapy. Malnutrition was defined as a body mass index (BMI) <18.5 kg/m² according to World Health Organization (WHO) criteria [14] and was further supported by biochemical markers, including low serum albumin and prealbumin levels. Patients with low BMI and/or reduced nutritional biomarkers were categorized as malnourished for analytical purposes. Baseline liver function tests, including alanine aminotransferase (ALT), aspartate aminotransferase (AST), bilirubin, and alkaline phosphatase (ALP), were performed before treatment initiation. Patients subsequently received standard first-line anti-tuberculosis therapy consisting of isoniazid, rifampicin, pyrazinamide, and ethambutol in accordance with national treatment guidelines.

Follow-up and hepatotoxicity assessment

Repeat clinical evaluations were performed at the same intervals as follow-up liver function tests, which were performed weekly throughout the first month and then at weeks 6 and 8. At week 4, nutritional biomarkers were re-evaluated to see if they correlated with elevated liver enzymes. ALT/AST >3× ULN with symptoms or >5× ULN without symptoms, coupled with verification of clinical characteristics such as nausea, vomiting, jaundice, and right upper quadrant discomfort, were considered indicators of drug-induced hepatotoxicity.

Statistical analysis

IBM SPSS Statistics for Windows, version 25.0 (IBM Corp., Armonk, New York, United States) was used to analyze the data. Frequencies and percentages were used for categorical data, while mean ± SD was used for continuous variables. To find independent predictors, factors significant in univariate analysis were incorporated into multivariate logistic regression, while the chi-square test was employed for categorical variables and the independent t-test for continuous data. In order to determine borderline or contributing risk, the relationship between dietary biomarkers and hepatotoxicity was assessed using logistic regression and Pearson correlation. Statistical significance was defined as a p-value of less than 0.05.

Control of Confounding Variables

To minimize confounding, all clinically relevant variables (age, gender, alcohol use, viral hepatitis status, diabetes mellitus, nutritional indicators, and baseline laboratory parameters) were first assessed in univariate analysis. Only variables with statistical significance (p < 0.05) or strong clinical relevance were entered into the multivariate logistic regression model. This stepwise adjustment approach was used to control for potential confounders and to identify independent predictors of drug-induced hepatotoxicity. Multicollinearity between variables was assessed prior to final model selection to ensure stability of estimates.

## Results

The study included 220 patients receiving first-line anti-TB therapy, among whom drug-induced hepatotoxicity occurred in 38 (17.27%), including 20 (9.09%) mild, 12 (5.45%) moderate, and six (2.73%) severe cases. Age distribution showed a higher proportion of hepatotoxicity among patients aged >45 years (18/38; 47.37%) compared with those ≤45 years (20/38; 52.63% in hepatotoxic vs 74.18% in non-hepatotoxic group), demonstrating a significant association with increased risk in older age (p=0.02). Female patients constituted 22 (57.89%) hepatotoxic cases versus 78 (42.86%) non-hepatotoxic cases, while male patients were relatively more frequent in the non-hepatotoxic group. Alcohol use showed a strong association with hepatotoxicity (12/38; 31.58% vs 18/182; 9.89%; p<0.001). Viral hepatitis (hepatitis B virus (HBV)/hepatitis C virus (HCV)) was also significantly higher in hepatotoxic patients (8/38; 21.05% vs 7/182; 3.85%; p<0.001). Diabetes mellitus and BMI-defined malnutrition showed no statistically significant association. Micronutrient deficiency was more frequent among hepatotoxic patients (12/38; 31.58% vs 28/182; 15.38%; p=0.019) (Table 2). Multivariate logistic regression was performed to adjust for potential confounding variables, thereby identifying independent associations between clinical and nutritional factors and hepatotoxicity.

**Table 2:** Clinical, nutritional, and laboratory predictors χ² = chi-square test statistic; *p < 0.05 is considered statistically significant. Reference category (OR = 1.00) indicates absence of exposure (e.g., ≤45 years, male gender, no alcohol use, no diabetes, no viral hepatitis, normal BMI, and normal micronutrient status). aOR: adjusted OR

Variable	Category	Hepatotoxicity (n=38), n (%)	No Hepatotoxicity (n=182), n (%)	χ² / t	p-value (Univariate)	OR / aOR	95% CI
Age	≤45 years	20 (52.63%)	135 (74.18%)	5.12	0.02*	1.00	—
	>45 years	18 (47.37%)	47 (25.82%)	5.12	0.02*	2.15	1.05–4.42
Gender	Male	16 (42.11%)	104 (57.14%)	2.87	0.09	1.00	—
	Female	22 (57.89%)	78 (42.86%)	2.87	0.09	1.87	1.01–3.47
Alcohol use	No	26 (68.42%)	164 (90.11%)	12.56	<0.001*	1.00	—
	Yes	12 (31.58%)	18 (9.89%)	12.56	<0.001*	3.42	1.50–7.79
Diabetes mellitus	No	28 (73.68%)	147 (80.77%)	0.98	0.32	1.00	—
	Yes	10 (26.32%)	35 (19.23%)	0.98	0.32	1.32	0.58–2.98
Viral hepatitis (HBV/HCV)	No	30 (78.95%)	175 (96.15%)	14.65	<0.001*	1.00	—
	Yes	8 (21.05%)	7 (3.85%)	14.65	<0.001*	5.28	1.87–14.85
BMI	≥18.5	29 (76.32%)	156 (85.71%)	2.07	0.15	1.00	—
	<18.5	9 (23.68%)	26 (14.29%)	2.07	0.15	1.45	0.65–3.21
Micronutrient status	Normal	26 (68.42%)	154 (84.62%)	5.53	0.019*	1.00	—
	Deficient	12 (31.58%)	28 (15.38%)	5.53	0.019*	2.01	1.06–3.81

Nutritional biochemical assessment demonstrated significantly reduced serum albumin and prealbumin in patients with hepatotoxicity. Serum albumin was lower in the hepatotoxic group (3.2 ± 0.4 g/dL) compared with non-hepatotoxic patients (3.8 ± 0.3 g/dL) (p<0.001). Similarly, prealbumin levels were reduced (17.5 ± 3.2 mg/dL vs 22.1 ± 3.5 mg/dL; p<0.001), indicating a significant deterioration in nutritional status among affected patients (Table 3). 

**Table 3:** Nutritional biomarkers (continuous variables) t = independent samples t-test; *p < 0.05 is considered statistically significant. aOR: adjusted odds ratio

Variable	Hepatotoxicity, mean ± SD	No Hepatotoxicity, mean ± SD	t-value	p-value	aOR	95% CI
Serum albumin (g/dL)	3.2 ± 0.4	3.8 ± 0.3	9.02	<0.001*	0.48	0.28–0.82
Prealbumin (mg/dL)	17.5 ± 3.2	22.1 ± 3.5	7.80	<0.001*	0.52	0.33–0.81

Biochemical analysis demonstrated markedly elevated liver enzymes in patients who developed drug-induced hepatotoxicity. The ALT and AST levels measured at the time of hepatotoxicity diagnosis during follow-up were significantly higher in the hepatotoxic group compared with patients without hepatotoxicity (ALT: 165.42 ± 50.31 U/L vs 42.18 ± 15.76 U/L; AST: 142.67 ± 45.20 U/L vs 38.45 ± 12.89 U/L; both p < 0.001). Similarly, total bilirubin (2.35 ± 0.98 vs 0.82 ± 0.25 mg/dL) and ALP (220.55 ± 70.41 vs 110.24 ± 40.31 U/L) were significantly elevated in the hepatotoxicity group (Table 4). Importantly, ALT and AST were components of the diagnostic criteria for drug-induced hepatotoxicity; therefore, their elevated levels reflect case definition and severity of hepatotoxicity rather than independent predictive associations.

**Table 4:** Comparison of liver function test parameters at the time of hepatotoxicity diagnosis between patients with and without drug-induced hepatotoxicity t = independent samples t-test. ALT and AST were part of the diagnostic criteria for drug-induced hepatotoxicity; therefore, their elevated values represent outcome confirmation and severity classification rather than independent predictive biomarkers. *p < 0.05 is considered statistically significant. ALT: alanine aminotransferase; AST: aspartate aminotransferase; ALP: alkaline phosphatase

Variable	Hepatotoxicity, mean ± SD	No Hepatotoxicity, mean ± SD	t-value	p-value
ALT (U/L)	165.42 ± 50.31	42.18 ± 15.76	14.96	<0.001*
AST (U/L)	142.67 ± 45.20	38.45 ± 12.89	14.10	<0.001*
Bilirubin (mg/dL)	2.35 ± 0.98	0.82 ± 0.25	9.56	<0.001*
ALP (U/L)	220.55 ± 70.41	110.24 ± 40.31	9.34	<0.001*

Longitudinal liver function and nutritional biomarker trends in all 220 patients showed gradual ALT and AST elevation during therapy, peaking at week 4 (ALT 50.15 ± 22.00 U/L, AST 47.22 ± 20.05 U/L) before slightly declining by week 8 (ALT 46.10 ± 20.22 U/L, AST 43.50 ± 18.05 U/L), as shown in Table 5. Bilirubin and ALP increased modestly (bilirubin 1.05 ± 0.48 mg/dL, ALP 130.50 ± 50.22 U/L at week 4). Serum albumin declined slightly during early therapy (3.8 ± 0.45 g/dL at baseline to 3.6 ± 0.38 g/dL at week 4), and prealbumin dropped from 21.5 ± 4.8 to 19.8 ± 3.8 mg/dL, reflecting transient nutritional impact during the intensive phase of TB therapy.

**Table 5:** Baseline and follow-up liver function tests with nutritional biomarkers (N=220) ALT: alanine aminotransferase; AST: aspartate aminotransferase; ALP: alkaline phosphatase

Time Point	ALT (U/L), mean ± SD	AST (U/L), mean ± SD	Bilirubin (mg/dL), mean ± SD	ALP (U/L), mean ± SD	Serum Albumin (g/dL), mean ± SD	Serum Prealbumin (mg/dL), mean ± SD
Baseline	38.45 ± 12.72	36.12 ± 11.54	0.81 ± 0.24	112.34 ± 41.20	3.8 ± 0.45	21.5 ± 4.8
Week 1	42.18 ± 15.31	39.45 ± 14.12	0.85 ± 0.28	115.20 ± 42.10	3.75 ± 0.42	21.0 ± 4.5
Week 2	45.22 ± 18.05	42.10 ± 16.33	0.92 ± 0.35	120.50 ± 45.35	3.7 ± 0.41	20.5 ± 4.2
Week 3	47.30 ± 20.11	44.55 ± 18.20	0.95 ± 0.40	125.10 ± 48.40	3.65 ± 0.40	20.2 ± 4.0
Week 4	50.15 ± 22.00	47.22 ± 20.05	1.05 ± 0.48	130.50 ± 50.22	3.6 ± 0.38	19.8 ± 3.8
Week 6	48.55 ± 21.50	45.80 ± 19.50	1.00 ± 0.44	128.30 ± 49.10	3.65 ± 0.40	20.1 ± 3.9
Week 8	46.10 ± 20.22	43.50 ± 18.05	0.98 ± 0.40	124.75 ± 47.60	3.7 ± 0.42	20.6 ± 4.1

Multivariate logistic regression for independent predictors of hepatotoxicity in 220 patients confirmed significant clinical and nutritional associations (Table 6). Age >45 years (aOR 2.15; 95% CI 1.05-4.42; p=0.036), female gender (aOR 1.87; p=0.045), alcohol use (aOR 3.42; p=0.003), viral hepatitis (aOR 5.28; p=0.002), low serum albumin <3.5 g/dL (aOR 2.12; p=0.041), and low prealbumin <20 mg/dL (aOR 2.45; p=0.014) were independent predictors, whereas diabetes mellitus (aOR 1.32; p=0.51) and BMI-defined malnutrition (aOR 1.45; p=0.36) were not significant.

**Table 6:** Multivariate logistic regression: independent predictors including nutritional biomarkers (N=220) * p < 0.05 considered statistically significant.

Variable	Adjusted Odds Ratio (aOR)	95% Confidence Interval (CI)	p-value
Age >45 years	2.15	1.05–4.42	0.036*
Female Gender	1.87	1.01–3.47	0.045*
Alcohol Use	3.42	1.50–7.79	0.003*
Viral Hepatitis	5.28	1.87–14.85	0.002*
Serum Albumin <3.5 g/dL	2.12	1.03–4.36	0.041*
Serum Prealbumin <20 mg/dL	2.45	1.20–5.00	0.014*
Diabetes Mellitus	1.32	0.58–2.98	0.51
Malnutrition (BMI <18.5 kg/m^2^)	1.45	0.65–3.21	0.36

## Discussion

The present study identified an incidence of drug-induced hepatotoxicity of 17.27%, which is consistent with previously reported rates ranging from 2% to 28% across different populations [15]. Earlier studies have demonstrated considerable variability in incidence due to differences in demographic profiles, clinical characteristics, and study settings, while a recent meta-analysis from India reported a pooled incidence of 12.6% [16]. The slightly higher incidence observed in our cohort may reflect regional factors such as nutritional status, comorbidities, and infectious burden, underscoring the need for population-specific risk evaluation.

Advanced age was associated with an increased risk of hepatotoxicity in our study, with 47.37% of affected patients being older than 45 years (aOR 2.15). This finding is consistent with previous reports identifying older age as a risk factor for anti-TB drug-induced hepatotoxicity, with earlier studies suggesting age thresholds above 35 years as a significant determinant [17,18]. The increased susceptibility in older individuals is likely related to reduced hepatic metabolic capacity and decreased drug clearance with advancing age.

Female sex was also associated with an increased risk of hepatotoxicity (57.89% vs 42.86%; aOR 1.87). This observation aligns with several studies reporting higher susceptibility among females, potentially due to hormonal influences and sex-related differences in drug metabolism [19]. However, contrasting evidence exists in the literature, where no significant gender association was observed, indicating heterogeneity across populations [17].

Significant associations were also observed between hepatotoxicity and both alcohol consumption and viral hepatitis. Alcohol use was present in 31.58% of hepatotoxic patients (aOR 3.42), while chronic viral hepatitis was identified in 21.05% (aOR 5.28). These findings are consistent with previous evidence demonstrating that alcohol-related liver injury and HBV/HCV co-infection increase susceptibility to anti-TB drug-induced hepatotoxicity due to pre-existing hepatic impairment and reduced metabolic reserve [20,21]. In the present study, alcohol use and viral hepatitis were compared against their respective reference groups (non-users and non-infected patients), supporting the assessment of their independent association with hepatotoxicity. These observations are in agreement with earlier studies highlighting alcohol consumption and viral hepatitis as major predisposing factors [20,21].

Nutritional status was also an important determinant of hepatotoxicity risk. Low serum albumin (<3.5 g/dL; aOR 2.12) and low prealbumin (<20 mg/dL; aOR 2.45) were significantly associated with hepatotoxicity. Similar findings have been reported previously, where hypoalbuminemia has been identified as an independent risk factor for drug-induced liver injury [18,19]. Additional evidence suggests that serum albumin levels below 3.5 g/dL are associated with increased vulnerability to hepatic injury, supporting the findings of the present study [22]. These results emphasize the role of nutritional status in drug metabolism and hepatic resilience.

Liver function test parameters measured at the time of hepatotoxicity diagnosis during follow-up were significantly higher in affected patients compared with those without hepatotoxicity (ALT 165.42 vs 42.18 U/L; AST 142.67 vs 38.45 U/L; p < 0.001). Importantly, ALT and AST were included as part of the diagnostic criteria for drug-induced hepatotoxicity in this study; therefore, their elevated levels reflect case definition and severity classification rather than independent predictive biomarkers of susceptibility. Similar observations have been reported in previous studies, where elevated transaminases were primarily interpreted as diagnostic indicators of hepatotoxicity rather than baseline risk predictors [18].

Longitudinal biochemical analysis of the overall cohort demonstrated a gradual increase in liver enzyme levels during anti-TB therapy, with peak mean ALT (50.15 U/L) and AST (47.22 U/L) observed at week 4, followed by a subsequent decline. These values represent overall treatment-related biochemical trends in the study population and are not specific to patients with hepatotoxicity. This temporal pattern is consistent with previous evidence indicating that anti-TB drug-induced liver enzyme elevations most commonly occur within the first month of therapy [22]. This highlights the importance of close biochemical monitoring during the early phase of treatment.

Study strengths and limitations

The strengths of the study are that it was prospective, incorporated a sufficient sample size (220 patients), and combined measurement of clinical and nutritional predictors of drug-induced hepatotoxicity in the initial phase of anti-TB therapy. The use of serum albumin, prealbumin, and micronutrient status enabled the use of a finer assessment of nutritional risk factors alongside conventional clinical and laboratory measurements. The intensive phase, which was followed up regularly every week, and longitudinal monitoring of liver function tests contributed to better early hepatotoxicity monitoring and recorded the dynamic changes in ALT, AST, bilirubin, and ALP. Limitations, however, are that BMI was a single anthropometric measurement of malnutrition, the assessment of other micronutrients other than vitamins B6 and B12 was not done, and genetic polymorphism or other host factors that may affect drug metabolism were not assessed. Besides this, the study was a single-center study and therefore, the regional differences might limit an extrapolation to larger populations.

## Conclusions

Drug-induced hepatotoxicity remains a clinically important complication among patients receiving first-line anti-TB therapy. Advanced age, female sex, alcohol consumption, viral hepatitis, and reduced nutritional biomarkers, particularly serum albumin and prealbumin levels, were associated with an increased risk of developing hepatotoxicity during treatment. These findings suggest that patients with these characteristics may require closer clinical and biochemical monitoring to facilitate early detection and management of hepatic adverse effects. Further large-scale studies are needed to confirm these associations and strengthen the evidence base.
